# Intergenerational transmission of parental smoking: when are offspring most vulnerable?

**DOI:** 10.1093/eurpub/ckac065

**Published:** 2022-07-17

**Authors:** J Alves, J Perelman, E Ramos, A E Kunst

**Affiliations:** NOVA National School of Public Health, Public Health Research Centre, CISP, NOVA University Lisbon, Lisbon, Portugal; NOVA National School of Public Health, Comprehensive Health Research Centre, CHRC, NOVA University Lisbon, Lisbon, Portugal; NOVA National School of Public Health, Public Health Research Centre, CISP, NOVA University Lisbon, Lisbon, Portugal; NOVA National School of Public Health, Comprehensive Health Research Centre, CHRC, NOVA University Lisbon, Lisbon, Portugal; Faculty of Medicine, Department of Public Health and Forensic Sciences, and Medical Education, University of Porto, Porto, Portugal; EPIUnit—Institute of Public Health, University of Porto, Porto, Portugal; Department of Public and Occupational Health, Amsterdam Public Health Research Institute, Amsterdam UMC, University of Amsterdam, Amsterdam, The Netherlands

## Abstract

**Background:**

Previous literature has showed that the likelihood of smoking is higher among offspring with smoking parents. The aim of this cohort study is to investigate during which smoking initiation stages and at what ages adolescents are more likely to be influenced by parental smoking.

**Methods:**

This study used the EPITeen Cohort, which recruited 13-year-old adolescents born in 1990, enrolled at schools in Porto, Portugal. Participants (*n* = 996) were followed across four waves at 13, 17, 21 and 24 years old. We computed the odds ratio (OR) and 95% confidence intervals for the prevalence of the different smoking states (never smoking, experimenter, less than daily smoker, daily smoker and former smoker), and incidence transitions between these states (to smoking experimenter; to less than daily smoker, to daily smoker; to former smoker) as function of age, parental smoking status and their interaction.

**Results:**

Compared with other participants, those with two smoking parents had an increased prevalence of experimentation at 13 years (OR for the interaction at 13 years compared with 24 years = 2.13 [1.50–3.01]) and daily smoking at all ages (OR for parental smoking =1.91 [1.52–2.40]). The latter increase is related to a significantly increased risk to transit from early smoking stages to daily smoking at all ages (OR for parental smoking = 1.83 [1.43–2.34]).

**Conclusions:**

Parental smoking influences offspring’s daily smoking prevalence especially by increasing the risk to transit to daily smoking up to early adulthood. Prevention should focus on parents and parental influences especially among offspring who may transition to daily smokers.

## Introduction

Although it is a preventable cause of death, 7 million people die annually worldwide due to tobacco use.^1^ Early smoking initiation is linked to higher use and lower cessation rates at adult ages.^2^ This fact is especially worrisome since data from 2007 to 2014 showed that around 9.8% of youths aged 13–15 years smoke cigarettes in European countries.^3^ In Portugal, according to the 2009/10 Health Behaviour in School-aged Children study, the smoking rate among 15-year olds was 10%.^4^

Previous evidence suggests that one of the factors contributing to youth smoking is parental smoking. Offspring with smoking parents have an increased risk of smoking initiation^5–7^ and life course smoking trajectories characterized by higher levels of smoking.^8–17^ Parental influences can be observed long after adolescence, including young adulthood,^18^^,^^19^ and until 30 or 40 years of age.^20–22^ According to Eurostat,^23^ in 2018 almost 90% of the 20–24 years old young adults still lived with their parents in Portugal, and 73% in the European Union.

Several authors^24–26^ have advances different explanations to explain this similarity in behaviour between parents and children: (i) parents may pass their preferences regarding tobacco consumption unto their children; (ii) parents are role models for their child, thus influencing their smoking behaviour by setting an example; and (iii) parents might ease children’s access to tobacco products by having cigarettes at home. Additionally, given the genetic influences on smoking (see Munafò and Johnstone^27^ for a review), parents and offspring may share genetic traits that influence addiction profiles and nicotine responses.

These influences may be critical at specific ages and at specific stages of the smoking initiation process. Such information may be important to inform smoking-prevention strategies that focus on particular ages and stages of smoking initiation. To date, however, there is only scarce evidence about the ages and stages when parental influences are strongest. Reliable evidence should come from longitudinal studies and the use of multiple respondents (parents and offspring). This study is the first to provide evidence from a European country. The aim of this study was to investigate how different stages of smoking were influenced by parental smoking, and at what ages this influence was most likely to occur.

## Methods

### Study design and population

This study used the Epidemiological Health Investigation of Teenagers in Porto (EPITeen) cohort, which started in 2003, and is described elsewhere.^28^ It is composed by adolescents born in 1990, studying in 55 schools of Porto (Portugal). The participants were followed across four waves: 2003/04, 2007/08, 2011/13 and 2014/15, when participants were on average 13, 17, 21 and 24 years old, respectively. The information was obtained through standardized self-reported questionnaires from parents and offspring. More details about the survey methods can be found in Supplementary appendix S1.

At the baseline in 2003, of the 2787 students eligible, 2159 agreed to participate. In the second wave, 1716 participants were re-evaluated. In the third and fourth waves, 1764 and 1094 participants were re-evaluated, respectively. We considered only those who had participated in the last wave (2014/15) and with at least more than two evaluations, and with information about parental smoking and education. The final sample was composed of 996 individuals.

The EPITeen Cohort was approved by the Portuguese Commission for Data Protection, and the Ethics Committees of Hospital S. João and of Instituto de Saúde Pública da Universidade do Porto (ISPUP). Written informed consent was obtained from legal guardians and adolescents in the 1st and 2nd waves, and from participants in the remaining waves.

### Measures

#### Offspring measures

Respondents were classified according to smoking prevalence in each wave. The never smokers were those who have never experienced cigarette smoking until that wave; the ever experimenters were those that have experimented with smoking at some point but have not smoked regularly until that wave (i.e. those who experimented were always experimenters thereafter, unless they started to smoke regularly); the less-than-daily smokers have smoked cigarettes in that wave but less than daily; the daily smokers smoked at least one cigarette per day at the time of that wave and the former smokers were those that reported smoking daily or less than daily in the past wave but not at the present wave (i.e. once they are former smokers, they are always former smokers, unless they start to smoke again).

We also created binary variables for smoking incidence. The incidence of experimenting was the new experimenters in that wave among the non-smokers in the previous wave. The incidence of less-than-daily smoking was the number of new less than daily smokers in the wave amongst those who did not smoke, experimented with smoking, smoked daily or quit smoking in the previous wave. Daily smoking incidence was the number of new daily smokers in the wave amongst those who did not smoke, experimented with smoking, smoked less than daily or quit smoking in the previous wave. Finally, former smoking incidence was the number of new former smokers in the wave among those who smoked daily or less than daily in the previous wave. The variables of incidence result from the transition from smoking states between the waves, and what occurred between the waves was not considered. For example, the number of participants that started and stopped smoking between those states was not included. Although the time of exposure was not exactly the same for all participants and between waves, to estimate incidence we assume that in every stage the respondents have the same duration of exposure to the risk factors.

#### Parental variables

Parental ever smoking was directly reported by parents, both in 2003 and 2007 waves, at 13 and 17 years old. Thus, we created a binary variable for having both parents smoking that equalled one if mother smoked (either in 2003 or 2007) and father smoked (either in 2003 or 2007), and equalled zero otherwise. The missing information for parental smoking was completed as follows: (i) when information was missing for both parents, they were dropped from the analysis; (ii) in cases information was reported for only one parent, we have completed with the parental smoking reported by the offspring in the questionnaire answered at school; (iii) if information was only reported for only one parent (e.g. single parent families), we considered that both parents smoked.

We also created a categorical variable for parental education based on the parent with the highest education level. Parental education was used in order to take into account the socioeconomic differences in parental smoking, since previous literature found a relation between education and smoking status in adults.^29^ Some categories had to be aggregated due to the lower number of observations in each category. The final variable had three categories: (i) primary and lower secondary education, (ii) secondary or post-secondary and (iii) tertiary education.

#### Analysis

Using a longitudinal design, we modelled whether the smoking behaviour was influenced by the parental smoking, in two steps. Stata 13.0 software was used to perform the analyses.

We first computed the estimated prevalence and incidence for each smoking status as function of parental smoking, using generalized estimating equation models (GEE), assuming a binomial distribution, and adjusting for sex and parental education, with fixed effects for age (which pertains to the wave of evaluation: at 13, 17, 21 and 24 years old). We then added interactions for age with parental smoking.

We then computed the odds ratio (OR), using GEE but also a binomial distribution and a logit link, for prevalence and incidence of smoking as function of parental smoking, adjusted by sex, parental education, age and with interactions for age with parental smoking.

Stratifying the analyses according to a detailed classification of parental smoking (e.g. one parent smoked, two parents smoked and neither parent smoked) would be challenging due to the low number of observations in some categories of parental smoking, and the low frequency of adolescent smoking at some ages. Nevertheless, a robustness check was performed, using the variable having at least one parent smoking (either in 2003 or 2007) instead of having both parents smoking.

## Results

Of the 996 participants, 48.70% were men (table 1). Regarding parental smoking, 48.0% had a mother that smoked, 68.8% had a father that smoked and 38.2% had both parents smoking.

**Table 1:** Descriptive statistics of the sample (EPITeen cohort, 2003, 2007, 2011 and 2014)

	All sample (%)	Parental smoking			
		Mother smoking (%)	Father smoking (%)	At least one parent smoking (%)	Having both parents smoking (%)
All sample	996 (100.0)	476 (48.0)	667 (68.8)	763 (76.6)	380 (38.2)
Sex					
Men	488 (48.7)	225 (46.4)	316 (66.7)	363 (74.4)	178 (46.8)
Parental education					
Primary and lower secondary	358 (26.4)	138 (38.8)	243 (71.3)	266 (74.3)	115 (30.3)
Secondary or post-secondary	282 (31.4)	137 (48.6)	198 (71.0)	219 (77.7)	116 (30.5)
Tertiary	356 (20.2)	201 (56.8)	226 (64.6)	278 (78.1)	149 (39.2)

The prevalence of smoking among the participants by age of evaluation is reported in table 2. At 13 years old (2013), 78.5% of the participants were never smokers and this value fell to 24.9% at 24 years old. The peak of the prevalence of ever experimenters and less-than-daily smokers was at 21 years old (37.0% and 8.9%), whereas the prevalence of daily smoking increased over time, but the highest increases were from 17 to 21 years old, still increasing up to 24 but more smoothly (24.6–26.2%). The percentage of formers smokers was low until 24 years old (7.3%). Table 2 also presents the matrix for the smoking transitions between the study waves.

**Table 2:** Prevalence for smoking measures and smoking transitions between the study waves (EPITeen cohort, 2003, 2007, 2011 and 2014)

Original state	New state						
	Never smoker	Experimenter	Smoke less than daily	Smoke daily	Former smoker	Total	%
Age 13–17						Age 13	
Never smoker	542	192	22	26	0	782	78.5
Experimenter	0	141	24	31	0	196	19.7
Smoke less than daily	0	0	4	5	3	12	1.2
Smoke daily	0	0	1	5	0	6	0.6
Former smoker	0	0	0	0	0	0	0.0
Age 17–21						Age 17	
Never smoker	281	174	30	57	0	542	54.4
Experimenter	0	195	40	98	0	333	33.4
Smoke less than daily	0	0	13	29	9	51	5.1
Smoke daily	0	0	5	60	2	67	6.7
Former smoker	0	0	1	1	1	3	0.3
Age 21**–**24						Age 21	
Never smoker	248	27	4	2	0	281	28.2
Experimenter	0	326	22	21	0	369	37.0
Smoke less than daily	0	0	26	32	31	89	8.9
Smoke daily	0	0	9	205	31	245	24.6
Former smoker	0	0	0	1	11	12	1.2
Total at age 24	248	353	61	261	73		
%	24.9	35.4	6.1	26.2	7.3		

The incidence of ever experimenting in participants with younger than 13 was 19.7% (table 3). Among the never smokers at 13, 24.6% ever experimented smoking from 13 to 17 years old; among never smokers at 17, 32.1% ever experimented between 17 and 21 years old; this incidence rate fell to 9.6% between 21 and 24 years old. The transition to less-than-daily smoking was the highest between 17 and 21 years (8.0%). The incidence of daily smoking was 6.3% between 13 and 17 years, 19.9% between 17 and 21 years old, and 7.5% between 21 and 24 years. Among smokers, the incidence of cessation was 16.7% 13–17 years old, 9.3% from 17 to 21 years and 18.6% from 21 to 24 years. Unadjusted prevalence and incidence measures stratified by parental smoking are presented in Supplementary appendix S2.

**Table 3:** Incidence rates for smoking measures (EPITeen cohort, 2003, 2007, 2011 and 2014)

	Age			
Offspring smoking incidence	<13	13–17	17–21	21–24
Experimentation				
New experimenters in the period (*N*)	196	192	174	27
Never smokers at the beginning of the period (*N*)	996	782	542	281
Incidence of experimentation (%)	19.7	24.6	32.1	9.6
Smoking less than daily				
New less than daily smokers in the period (*N*)	12	47	76	35
Never smokers, experimenters, daily smokers and former smokers at the beginning of the period (*N*)	996	984	945	907
Incidence of less than daily smoking (%)	1.2	4.8	8.0	3.9
Smoking daily				
New daily smokers in the period (*N*)	6	62	185	56
Never smokers, experimenters, less-than-daily smokers and former smokers at the beginning of the period (*N*)	996	990	929	751
Incidence of daily smoking (%)	0.6	6.3	19.9	7.5
Former smoking				
New former smokers in the period (*N*)	.	3	11	62
Smokers at the beginning of the period (*N*)	.	18	118	334
Incidence of former smoking (%)	.	16.7	9.3	18.6

**Figure 1:**
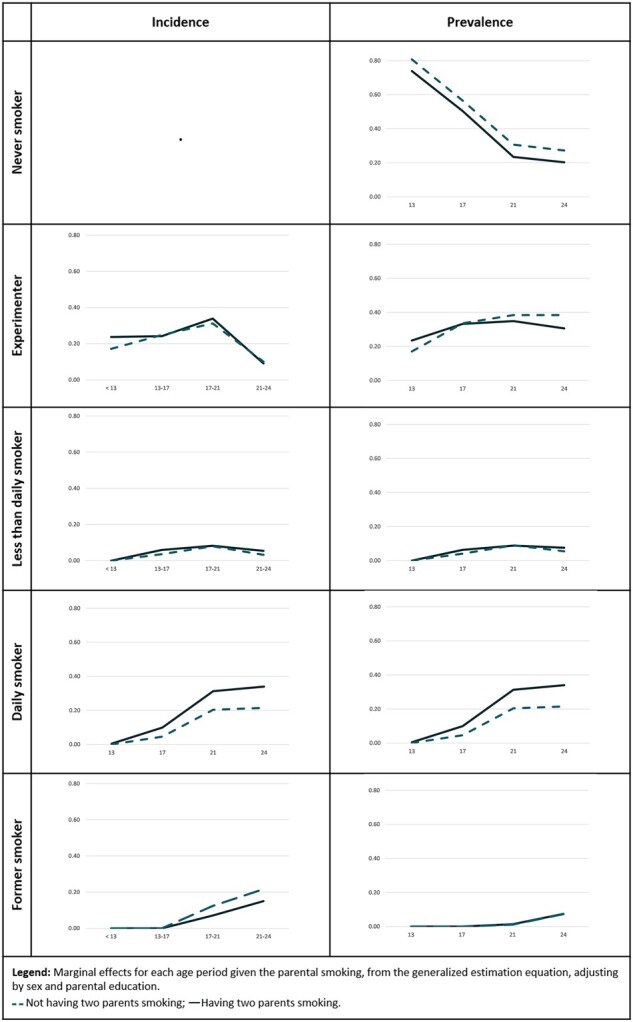
Smoking incidence and prevalence trends by parental smoking status, adjusting for sex and parental education, with fixed effects for age (EPITeen cohort 2003, 2007, 2011 and 2014).

Figure 1 presents the adjusted prevalence and incidence according to parental smoking. The prevalence of never smoking was lower amongst those who had both parents smoking, but the age-related decline was similar for those with and without smoking parents. The incidence of ever experimenting before 13 years old was higher among those who had both parents smoking, but not after that age. Likewise, the prevalence of ever experimenting at 13 years of age was higher for the ones that had both parents smoking at 13 years old, but not at higher ages. Daily smoking incidence was higher for those who had both smoking parents than those who did not. This difference emerged in the 13–17 years period and persisted thereafter. As a result of these transitions, the prevalence of daily smoking increased in both groups, but was higher among those who had smoking parents. No large differences between these two groups were observed with regards to less-than-daily smoking and former smoking.

Table 4 presents the OR from the GEE analyses, for prevalence and incidence of smoking. Model 1 is adjusted by sex, parental education, age and parental smoking, while Model 2 also included interactions between age parental smoking. Having both parents smoking almost doubled the likelihood of being daily smoker (OR = 1.91; 95% CI [1.52–2.40]). The interaction with age was significant for the prevalence of ever experimenting (*P* < 0.001), indicating a stronger association with parental smoking amongst those 13 and 17 years old than those 24 years old. For the other variables the interaction with age was not significant. Regarding incidence, having both smoking parents was associated with an increased likelihood of ever experimenting smoking (OR = 1.18; 95% CI [0.97–1.43]), becoming a less than daily smoker (OR = 1.18; 95% CI [0.86–1.61]) and becoming daily smoker (OR = 1.83; 95% CI [1.43–2.34]), although this association was statistically significant only for the transition to daily smoking (*P* < 0.05).

**Table 4:** Adjusted OR for the likelihood of smoking prevalence and incidence over time (EPITeen cohort, 2003, 2007, 2011 and 2014)

	Never smoker	Experimenter	Less than daily smoker	Daily smoker	Former smoker
Prevalence					
Model 1					
Two smoking parents	0.73 [0.58–0.92]	0.91 [0.75–1.11]	1.13 [0.82–1.55]	1.91 [1.52–2.40]	1.02 [0.62–1.66]
Model 2					
13 years old × smoking parents	0.98 [0.73–1.32]	2.13 [1.50–3.01]	0.57 [0.16–2.05]	0.88 [0.19–4.13]	NE
17 years old × smoking parents	1.13 [0.86–1.49]	1.40 [1.01–1.92]	0.88 [0.42–1.85]	1.20 [0.73–1.96]	1.15 [0.39–3.39]
21 years old × smoking parents	1.02 [0.76–1.35]	1.21 [0.88–1.66]	0.67 [0.35–1.30]	0.94 [0.67–1.32]	1.15 [0.39–3.39]
24 years old × smoking parents^a^	1.00	1.00	1.00	1.00	1.00
*P*-value for interaction	0.708	0.000	0.611	0.809	0.804
Incidence					
Model 1					
Two smoking parents		1.18 [0.97–1.43]	1.18 [0.86–1.61]	1.83 [1.43–2.34]	0.64 [0.37–1.08]
Model 2					
<13 years old × smoking parents		1.72 [0.68–4.33]	0.52 [0.13–2.09]	0.82 [0.15–4.43]	NE
13–17 years old × smoking parents		1.10 [0.44–2.80]	0.85 [0.35–2.10]	1.20 [0.57–2.53]	NE
17–21 years old × smoking parents		1.29 [0.50–3.32]	0.65 [0.28–1.50]	0.79 [0.42–1.48]	1.20 [0.31–4.65]
21–24 years old × smoking parents^a^		1.00	1.00	1.00	1.00
*P*-value for interaction		0.247	0.672	0.583	0.794

Model 1: adjusted for age, sex and parental education. Model 2: adjusted for age, sex, parental education and interactions of age periods with having both parents smoking.

a Reference category. NE = Could not be estimated due to small number of participants at risk.

Finally, a robustness check was performed, using the variable having at least one parent smoking in either 2003 or 2007 (at 13 or 17 years old) instead of having both parents smoking. The same patterns were observed for most of the smoking states, except for daily smoking, for which the convergence could not be achieved. The results can be found in Supplementary appendix S3. It is common practice in the literature to provide the analyses stratified by sex. However, as we found that the results were quite similar for men and women, the main text only presents results for the population as a whole to increase the statistical power of the analyses and avoid redundancy of presentation. Supplementary appendix S4 presents the results stratified by sex.

## Discussion

### Key results

The results showed that having smoking parents increases the offspring’s risk of experimentation in early adolescence, and the risk of transitioning to daily smoking after experimentation until early adulthood. The increase in risk for other transitions was generally small and not statistically significant.

### Evaluation of potential limitations

The longitudinal study is subject to attrition over time. The attrition rate over time in this survey was 39%. This may affect the representativeness of the study population, as it reduced the sample size. Due to the small sample, we may have been unable to demonstrate with statistical significance some of the expected associations between parental smoking and smoking-related transition rates.

Of all participants, 38% had at least one missing answer about smoking, either because they refused to answer or because they were not evaluated in a specific wave. We addressed this latter problem by using the age of smoking initiation and the age of trying the first cigarette as indicated in the next wave, in order to reconstruct a smoking history for participants lacking only one response in all four surveys. This allowed us to recover 304 participants.

It was not possible to evaluate the effect of dose-exposure according to the number smoking parents, and the same-sex influences. We had to stratify the analysis between smoking statuses, and to add interactions between parental smoking and year. This would have left some categories without a sufficient number of cases. For example, in 2007 only 6% of those who smoke daily had one of the parents smoking. Therefore, we performed a robustness check, using the variable having at least one parent smoking (either in 2003 or 2007) instead of having both parents smoking. The same patterns were observed for most of the smoking states.

Finally, we would like to emphasize that these results were obtained in the specific context of a Southern European country. Due to differences in culture and the staging of the smoking epidemic, the parental influence as observed in Portugal may be different elsewhere.

### Interpretation of results

Offspring with both smoking parents had an increased risk of experimentation before the age of 17 years. Having smoking parents could imply easy access to tobacco products at home^26^ and this might prompt them to experiment with smoking. Parents can also pass their preferences regarding consumption to children in early adolescence, e.g. by showing the social function of cigarette consumption, and suggesting a positive utility by smoking in front of them.^25^

The risk of transit from smoking experimentation to daily smoking was greater among those who had smoking parents. The offspring observe their parents’ smoking behaviours and form their own attitudes, beliefs and behaviours from that observation.^30^ Offspring that observe parental smoking might perceive smoking as an acceptable social behaviour.^31^ Additionally, parental role models are transmitting contradictory messages regarding the health risks of smoking, because they smoke despite all the warnings that smoking is harmful. As a result, offspring might underestimate the future costs of smoking when making decisions about their own smoking behaviour.^32^

The influence of parental smoking was less in magnitude for smoking experimentation than for daily smoking. Other factors might be more influential for experimentation between 13 and 17 years, such as peer encouragement. Flay et al.^33^ argued that friends have a particular influence on the transition to experimental use, through smoking approval and cigarette offers, whereas the family context was more important to the transition to regular use.

The prohibition of tobacco sales to minor might also contribute to the large difference in daily smoking between those with and without smoking parents between 17 and 21. Despite the evidence that bans on sales to minors are weakly enforced in Portugal, the access to cigarettes just before and after legal age may be greater among families with smoking parents.^34^^,^^35^

The importance of genetic influences regarding smoking is well established. Studies using twins showed that genetics explain ∼50% of smoking behaviour, and that, once started, smoking can be maintained through genetic predisposition.^36^^,^^37^ Parents can share with offspring the same susceptibility to addiction to nicotine, and the same response to pharmacotherapy.^27^^,^^38^ An important role of genetics is congruent with our finding that the influence of parental smoking was greatest on the risk to transition from early smoking stages towards daily smoking, and that this increased risk was maintained until young adult age.

## Conclusions

Parents influence offspring’s daily smoking prevalence by increasing the risk of experimentation in early adolescence, and especially by increasing the risk of offspring’s daily smoking after experimentation. Parental smoking influences should be taken into account in policies and programs that seek to prevent older adolescents from transitioning from experimenters to daily smokers.

## Supplementary data

Supplementary data are available at *EURPUB* online.

## Funding

This study received funding from the Foundation for Science and Technology—FCT (Portuguese Ministry of Science, Technology and Higher Education) Unidade de Investigação em Epidemiologia—Instituto de Saúde Publica da Universidade do Porto (EPIUnit) (UIDB/04750/2020). This study is also part of the SILNE-R project, which received funding from the European Commission (EC), Horizon2020 Program, Call PHC6-2014, under Grant Agreement no. 635056. The present publication was also funded by Fundação Ciência e Tecnologia, IP national support through CHRC (UIDP/04923/2020).

*Conflicts of interest*: None declared.

Key points

- To prevent smoking among this offspring, it is important to identify the life periods where they are at an increased risk to engage this behaviour.
- This study provides evidence about the ages and stages when parental influences are strongest, using a longitudinal design and multiple respondents (parents and offspring).
- Prevention should focus on parents and parental influences, especially among offspring who may transit to daily smokers.

## Supplementary Material

ckac065_Supplementary_Data
