# Supplementary material for: Intergenerational transmission of parental smoking: when are offspring most vulnerable?
Source: Eur J Public Health. 2022 Jul 17;32(5):741–6. doi: 10.1093/eurpub/ckac065 (PMC9527976; doi:10.1093/eurpub/ckac065)
Supplement: ckac065_Supplementary_Data [file ckac065_supplementary_data.pdf]

Appendix 1 > Methods:

Epidemiological Health Investigation of Teenagers in Porto (EPITeen) started in the 2003/2004 school year. It is a population-based cohort of urban adolescents born in 1990 and enrolled in a school in Porto. School boards of all public and private Porto schools were approached. All public schools and 79% of private schools agreed to participate and provide the students' contact information. At the baseline, of the 2,787 students eligible (2126 from public and 662 from private schools), 2,159 agreed to participate. This yielded a 77.5% participation rate (similar in public and private schools). Besides the baseline, three more waves were performed, having on average: 13 years old (2003/2004), 17 years old (2007/2008), 21 years old (2011/2013), and 24 years old (2014/2015. In the second wave, 1,716 participants were re-evaluated. In the third and fourth study waves, 1,764 and 1,094 participants were re-evaluated, respectively.

Standardized procedures were performed throughout all waves. The first and second evaluation was performed at school and the third and the fourth in our Department at Porto Medical School. The evaluation comprises a physical examination performed by a trained team and self-administered questionnaires. One questionnaire was completed at home and comprises information on demographic and social characteristics, family history of disease and all data regarding mother and father (including smoking habits); and another self-administered questionnaire was completed at school/department regarding participants behaviours. In the first and second wave the questionnaire completed at school also included questions regarding parental smoking.

The EPITeen Cohort was approved by the Portuguese Commission for Data Protection, and the Ethics Committees of Hospital S. João and of Instituto de Saúde Pública da Universidade do Porto (ISPUP). Parents received written information about the study design and objectives. Written informed consent was obtained from parents and adolescents in the first and second waves, and from participants in the remaining waves.

For the analyses, we considered only those who participated in the last wave (2014/2015) and with at least more two evaluations, and with information about parental smoking and profession. The final sample was composed of 996 individuals. The table below shows the distribution of the observations. The observations

dropped from the analysis were fairly distributed between sex and more concentrated among the lowest parental education.

	Retained n (%)	Dropped n (%)	Total n (%)
**Sex**	**996**	**1,946**	**2,942**
Women	508 (51.0)	999 (51.3)	1,507 (51.2)
Men	488 (49.0)	947 (48.7)	1,435 (48.8)
**Highest parental education**	**996**	**1,946**	**2,942**
Primary and lower secondary	358 (35.9)	976 (50.3)	1,337 (45.5)
Secondary or post-secondary	282 (28.3)	449 (23.1)	731 (24.9)
Tertiary	356 (35.7)	405 (20.8)	761 (25.9)
Missing	0 (0.0)	113 (5.8)	113 (3.8)

The offspring were questioned about smoking patterns, using the following questions:

- Do you smoke or ever smoked? (1 = yes; 0 = no)
- How do you smoke? (1 = experimented, but do not smoke; 2 = smoked, but not every day; 3 = smoke at least a cigarette a day)

The parental smoking habits were evaluated only in the first and second evaluations (2003 and 2007). The questions used for this study were the following:

- Father: Do you smoke or ever smoked? (1 = yes; 0 = no)
- Mother: Do you smoke or ever smoked? (1 = yes; 0 = no)

Appendix 2 > Unadjusted prevalence and incidence measures, stratified by parental smoking.

	Age			
**Offspring smoking prevalence (%)**	**13**	**17**	**21**	**24**
**All sample**				
Never smoker	78.5	54.4	28.2	24.9
Experimenter	19.7	33.4	37.1	35.4
Smoke less than daily	1.2	5.1	8.9	6.1
Smoke daily	0.6	6.7	24.6	26.2
Former smoker	0.0	0.3	1.2	7.3
**Having both parents smoking**				
Never smoker	74.5	50.8	24.0	20.8
Experimenter	23.7	33.2	34.7	30.5
Smoke less than daily	1.1	5.8	8.7	7.4
Smoke daily	0.8	10.0	31.3	34.0
Former smoker	0.0	0.3	1.3	7.4
**Not having both parents smoking**				
Never smoker	81.0	56.7	30.8	27.4
Experimenter	17.2	33.6	38.5	38.5
Smoke less than daily	1.3	4.7	9.1	5.4
Smoke daily	0.5	4.7	20.5	21.4
Former smoker	0.0	0.3	1.1	7.3
**Offspring smoking incidence (%)**	**<13**	**13-17**	**17-21**	**21-24**
**All sample**				
Experimentation	19.7	24.6	32.1	9.6
Smoking less than daily	1.2	4.8	8.0	3.9
Smoking daily	0.6	6.3	19.9	7.5
Former smoking	.	16.7	9.3	18.6
**Having both parents smoking**				
Experimentation	23.7	24.0	33.7	8.8
Smoking less than daily	1.1	5.6	8.1	4.9
Smoking daily	0.8	9.6	24.6	10.7
Former smoking	.	14.3	8.3	15.1
**Not having both parents smoking**				
Experimentation	17.2	24.9	31.2	10.0
Smoking less than daily	1.3	4.3	8.0	3.2
Smoking daily	0.5	4.2	17.2	5.7
Former smoking	.	18.2	10.3	21.4

Appendix 3 > Adjusted odds ratio for the likelihood of smoking prevalence and incidence over time (EPITeen cohort, 2003, 2007, 2011, and 2014).

	Never smoker		Experimenter		Less than daily smoker		Daily smoker		Former smoker	
**Prevalence**										
Model 1										
One smoking parent	0.60	[0.46; 0.78]	1.23	[0.98; 1.54]	0.93	[0.65; 1.34]	1.63	[1.23; 2.16]	1.18	[0.66; 2.13]
Model 2										
13 years old x one smoking parent	0.86	[0.60; 1.24]	1.73	[1.12; 2.67]	0.89	[0.22; 3.62]	CNA		NE	
17 years old x one smoking parent	0.87	[0.64; 1.18]	1.46	[1.01; 2.12]	0.97	[0.41; 2.29]	CNA		NE	
21 years old x one smoking parent	1.02	[0.75; 1.39]	0.99	[0.69; 1.42]	0.79	[0.37; 1.67]	CNA		0.76	[0.22; 2.62]
24 years old x one smoking parent [(1)]	1.00		1.00		1.00		CNA			
P-value for interaction	0.633		0.017		0.925		CNA		0.665	
**Incidence**										
Model 1										
One smoking parent	.		1.55	[1.23; 1.95]	0.94	[0.65; 1.34]	1.76	[1.28; 2.43]	0.90	[0.47; 1.74]
Model 2										
<13 years old x one smoking parent	.		1.21	[0.43; 3.40]	1.07	[0.23; 4.93]	CNA		NE	
13-17 years old x one smoking parent	.		1.07	[0.38; 2.98]	1.16	[0.41; 3.30]	CNA		NE	
17-21 years old x one smoking parent	.		0.86	[0.31; 2.42]	1.05	[0.40; 2.71]	CNA		0.72	[0.12; 4.17]
21-24 years old x one smoking parent [(1)]	.		1.00		1.00		CNA			
P-value for interaction	.		0.722		0.993		CNA		0.715	

Legend: Model 1 - Adjusted for age, sex and parental education. Model 2 - Adjusted for age, sex, parental education, and interactions of age periods with having both parents smoking. [(1)] Reference category. NE = Could not be estimated due to small number of participants at risk. CAN = convergence not achieved.

Appendix 4 > Stratification by sex:

	Never smoking		Experimenting		Less than daily		Daily		Former smoking	
Prevalence										
**WOMEN**										
Model 1										
Two smoking parents	0.66	[0.48; 0.91]	0.86	[0.66; 1.14]	1.44	[0.87; 2.36]	2.31	[1.64; 3.25]	1.30	[0.67; 2.50]
Model 2										
Two smoking parents	0.69	[0.46; 1.02]	0.65	[0.45; 0.95]	1.28	[0.58; 2.84]	2.35	[1.52; 3.65]	1.28	[0.66; 2.47]
13yo x SP	0.86	[0.60; 1.24]	2.51	[1.60; 3.94]	1.74	[0.27; 11.32]	0.43	[0.08; 2.35]		
17yo x SP	0.96	[0.68; 1.35]	1.56	[1.02; 2.39]	1.79	[0.58; 5.57]	0.93	[0.49; 1.79]		
21yo x SP	1.02	[0.72; 1.46]	1.07	[0.70; 1.63]	0.93	[0.35; 2.50]	0.89	[0.54; 1.44]	2.43	[0.49; 12.05]
24yo x SP										
**MEN**										
Two smoking parents	0.81	[0.58; 1.12]	0.97	[0.74; 1.27]	0.93	[0.61; 1.41]	1.57	[1.16; 2.14]	0.72	[0.34; 1.56]
Model 2										
Two smoking parents	0.64	[0.40; 1.04]	0.77	[0.52; 1.15]	1.50	[0.75; 3.00]	1.69	[1.15; 2.49]	0.75	[0.35; 1.61]
13yo x SP	1.28	[0.79; 2.10]	1.70	[0.98; 2.92]	0.19	[0.02; 1.69]				
17yo x SP	1.45	[0.93; 2.27]	1.24	[0.77; 2.01]	0.54	[0.20; 1.48]	1.36	[0.64; 2.89]		
21yo x SP	1.03	[0.64; 1.65]	1.39	[0.87; 2.23]	0.53	[0.22; 1.29]	0.92	[0.57; 1.48]	0.46	[0.06; 3.55]
24yo x SP										

	Experimenting		Less than daily		Daily		Former smoking	
Incidence								
**WOMEN**								
Model 1								
Two smoking parents	1.21	[0.92; 1.58]	1.59	[0.98; 2.58]	2.04	[1.41; 2.94]	0.70	[0.34; 1.45]
Model 2								
Two smoking parents	0.84	[0.25; 2.85]	1.33	[0.47; 3.75]	1.89	[0.73; 4.89]	0.60	[0.27; 1.33]
13yo x SP	1.95	[0.54; 7.10]	1.67	[0.21; 13.35]	0.54	[0.07; 4.04]		
17yo x SP	1.14	[0.31; 4.28]	2.12	[0.50; 9.03]	1.36	[0.43; 4.35]		
21yo x SP	1.21	[0.32; 4.63]	0.90	[0.26; 3.21]	1.02	[0.36; 2.93]	2.56	[0.38; 17.11]
24yo x SP								
**MEN**								
Two smoking parents	1.18	[0.97; 1.43]	1.18	[0.86; 1.61]	1.83	[1.43; 2.34]	0.64	[0.37; 1.08]
Model 2								
Two smoking parents	0.88	[0.37; 2.08]	1.55	[0.79; 3.07]	2.01	[1.17; 3.44]	0.62	[0.35; 1.10]
13yo x SP	1.72	[0.68; 4.33]	0.52	[0.13; 2.09]	0.82	[0.15; 4.43]		
17yo x SP	1.10	[0.44; 2.80]	0.85	[0.35; 2.10]	1.20	[0.57; 2.53]		
21yo x SP	1.29	[0.50; 3.32]	0.65	[0.28; 1.50]	0.79	[0.42; 1.48]	1.20	[0.31; 4.65]
24yo x SP								

Note: the interactions between sex and parental smoking were not significant for all the prevalence and incidence variables.
